# Acknowledgement to Reviewers of *Genes* in 2013

**DOI:** 10.3390/genes5010106

**Published:** 2014-02-27

**Authors:** Genes Editorial Office

**Affiliations:** MDPI AG, Klybeckstrasse 64, CH-4057 Basel, Switzerland

The editors of *Genes* would like to express their sincere gratitude to the following reviewers for assessing manuscripts in 2013:

Afelik, Solomon
Aguileta, Gabriela
Araki, Ryoichi
Benkovic, Stephen J.
Benson, M. Douglas
Bergmann, Jan H.
Bianchetti, Laurent
Bishop, Cleo L.
Bjergbæk, Lotte
Boizet-Bonhoure, Brigitte
Bull, Susan
Burgess, Robert W.
Capel, Blanche
Casati, B.
Challiss, R.A. John
Chami, Mounia
Chang, C.-C.
Chen, Jeremy J.W.
Chénais, Benoît
Chrast, Roman
Clark, Andy
Cook, Mathew
Crews, David
Dion, Vincent
Doetsch, Paul W.
Domingos, Pedro M.
Dreyer, Jean-luc
Driver, John
Efrat, Himon
Ellard, S.
Gaerstel, Matthias
Gascon, Eduardo
Gomer, Richard H.
Gómez, María
Greenberg, Narayan
Grimpe, Barbara
Harris, Reuben
Haynes, Cole M.
Hellstrand, S.
Hetz, Claudio
Hingorani, Manju
Isaacs, Aaron
Ishiura, Shoichi
Jiravanichpaisala, Pikul
Johnson, Edward
Josyula, Ratnakar
Kaminska, Bozena
Kennerson, Marina L.
Klungland, Arne
Krau, Jeffrey A.
Kuiper, Roland
Landolph, Joseph R.
Lang, Andrew S.
Laybutt, Ross
Liu, Yuan
Long, Quan
Lynch, Henry T.
Mahajan, Supriya D.
Matsuo, Koichi
McCormick, Jennifer B.
Moltó-Puigmartí, Carolina
Mooyaart, A.L.
Morrione, Andrea
Mutch, David
Myre, Michael
Niggli, Ernst
Noguchi, Eishi
O'donnell, Kathryn A.
Owen, Katharine R.
Panda, Debasis
Parker, Lisa
Pask, Andrew J.
Paterson, Andrew
Pavlov, Youri I.
Platzer, Matthias
Povirk, Lawrence F.
Powis, S.J.
Rey, Rodolfo A.
Ribeiro, Andre S.
Ruat, Martial
Salnikow, Konstantin
Schepers, Kenneth S.
Shiotsuki, Takahiro
Sleiman, Patrick M.A.
Sonntag, Kai-christian
Soranzo, Nicole
Sørensen, Claus Storgaard
Speck, Christian
Størling, Joachim
Takisawa, Haruiko
Valenzuela, Manuel S.
Van Coppenolle, Fabien
Vashee, Sanjay
Vaxillaire, Martine
Wahlestedt, Claes
Wang, Han-Ching
Wei, Zong
Weinreich, Michael
Weterman, M.A.J.
Winqvist, Robert
Yuan, Qipeng
Zeng, Xiankun
Zhang, Qin
Zhang, Zhiguo
Zhao, Feng-qi
Zhao, Xiaolan
Zhu, Biao

